# Targeting dual specificity protein kinase TTK attenuates tumorigenesis of glioblastoma

**DOI:** 10.18632/oncotarget.23152

**Published:** 2017-12-11

**Authors:** Jia Wang, Yuchen Xie, Xiaobin Bai, Ning Wang, Hai Yu, Zhong Deng, Minxue Lian, Shuo Yu, Hao Liu, Wanfu Xie, Maode Wang

**Affiliations:** ^1^ Department of Neurosurgery, The First Affiliated Hospital of Xi’an Jiaotong University, Xi’an, Shaanxi 710061, China; ^2^ School of Medicine, Xi’an Jiaotong University, Xi’an, Shaanxi 710061, China

**Keywords:** glioma stem-like cells, glioblastoma, TTK, MTFR2

## Abstract

Accumulating evidence has proved that glioma stem-like cells (GSCs) are responsible for tumorigenesis, treatment resistance, and subsequent tumor recurrence in glioblastoma (GBM). In this study, we identified dual specificity protein kinase TTK (TTK) as the most up-regulated and differentially expressed kinase encoding genes in GSCs. Functionally, TTK was essential for *in vitro* clonogenicity and *in vivo* tumor propagation in GSCs. Clinically, TTK expression was highly enriched in GBM, moreover, was inversely correlated with a poor prognosis in GBM patients. Mechanistically, mitochondrial fission regulator 2 (MTFR2) was identified as one of the most correlated genes to TTK and transcriptionally regulated TTK expression via activation of TTK promoter. Collectively, MTFR2-dependent regulation of TTK plays a key role in maintaining GSCs in GBM and is a potential novel druggable target for GBM.

## INTRODUCTION

Glioblastoma (GBM) is the most common intra-parenchymal and lethal brain cancer and patients are left behind without any curable therapy to date [1–3]. The current standard therapy for GBM includes maximal surgical resection followed by radiotherapy and chemotherapy [3]. Despite the advances, these treatment strategies fails to eliminate a subset of tumor cells that escape from therapeutic insult and result in tumor recurrence, leading to reduced survival in these patients [4, 5]. A small population of GBM cells which shows the similar characteristics to normal stem/progenitor cells with unlimited proliferation and self-renewal capacity, termed as glioma stem-like cells (GSCs), could promote the resistance of radiotherapy and chemotherapy then drive tumor invasion and recurrence of GBM [6, 7]. Therefore, there is an urgent need to deeply investigate the molecular signaling pathways of tumorigenesis and invasion in GSCs.

Genes for dual specificity protein kinase TTK (TTK), or named monopolar spindle 1 (MPS1), was located on chromosome 6q13-6q21 and encodes a phosphorylated protein kinase on serine, threonine, and tyrosine [8]. TTK function is associated with cell proliferation and is essential for chromosome alignment by enhancing aurora kinase B (AURKB) activity via direct phosphorylation at the centromere [9]. Recent studies indicates that elevated TTK expression leads to tumorigenesis and poor prognosis in multiple types of malignant cancer, including gastric cancer, pancreatic cancer, breast cancer and liver cancer and bladder cancer, *et al* [10–14]. However, it is ambiguous about expression and functions of TTK in GBM, especially in GSCs. Furthermore, the up-stream regulating mechanism of TTK still remains unclear. Altogether, detailed studies focusing on the expression, function, and regulation mechanism of TTK in GBM and GSCs are needed.

Mitochondrial fission regulator 2 (MTFR2), also termed as family with sequence similarity 54, member A (FAM54A), is a poorly investigated protein in human cancer. MTFR2 belongs to the MTFR1/FAM54 family and 2 isoforms of MTFR2 are produced by alternative splicing [15]. Additionally, MTFR2 plays an important functional role in mitochondrial, aerobic respiration and promotes mitochondrial fission in eukaryotic animal cells [15]. Despite these findings, there is very limited amount of evidence which investigates MTFR2 functions in tumor.

## RESULTS

### TTK expression was highly enriched in GSCs

**Figure 1:**
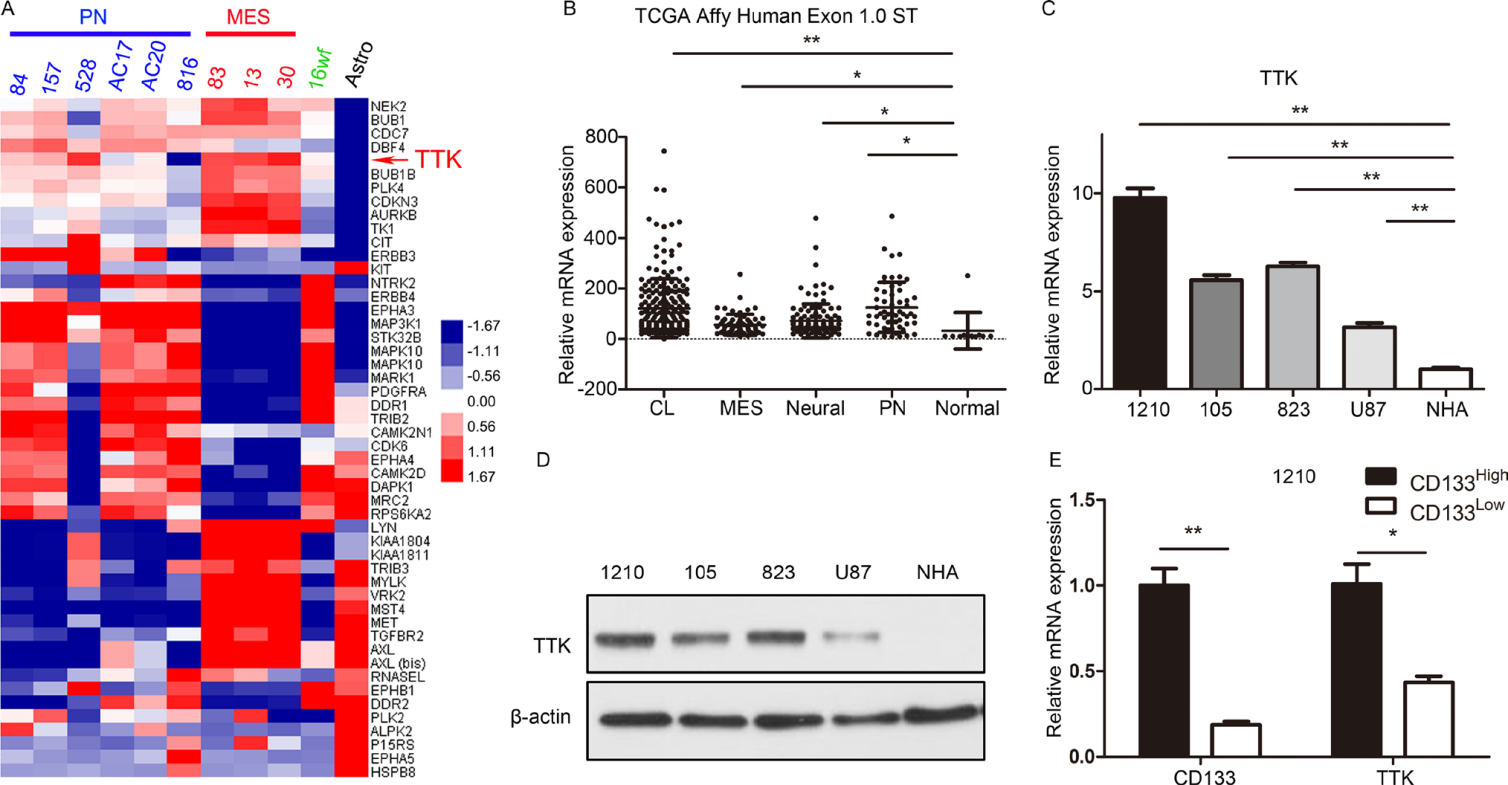
TTK expression was highly enriched in GSCs (**A**) Genome-wide transcriptome microarray analysis from Mao’s database showed that TTK was one of the most up-regulated kinase encoding genes in GSCs samples compared to normal tissue. (**B**) Analysis of TCGA database indicated that TTK was highly expressed in all the 4 subgroups of GBM (classical, CL; mesenchymal, MES; neural and proneural, PN) compared with non-tumor tissue (^*^P < 0.05, ^**^P < 0.01, with one-way ANOVA followed by Dunnett’s post-test, platform: Affy Human Exon 1.0 ST). (**C**) qRT-PCR analysis showed TTK mRNA expression was elevated in 3 GSCs cell lines (1210, 105, 823) compared with GBM U87 cells and normal astrocytes (NHA) (*n* = 3, ^**^P < 0.01, with one-way ANOVA followed by Dunnett’s post-test). (**D**) Western blotting analysis indicated that TTK protein expression was enriched in3 GSCs cell lines (1210, 105, 823) compared with GBM U87 cells and normal astrocytes (NHA). β-actin served as a control. (**E**) qRT-PCR analysis showed TTK expression was elevated in GSCs enriched for the CD133 surface marker relative to matched negative tumor cells from 1210 glioma sphere lines (*n* = 3,^*^P < 0.05, ^**^P < 0.01, with one-way ANOVA followed by Dunnett’s post-test).

To identify the functional role of TTK in GBM, an bioinformatics analysis based on the microarray database published in 2013 [16] was performed and the results indicated that TTK was one of the most elevated kinase-encoding genes in GSCs-containing cell lines when compared with normal human astrocyte cells (Figure 1A). Additionally, we found that TTK expression was dramatically up-regulated in all the 4 subgroups of GBM (classical, mesenchymal, neural and proneural) according to TCGA database compared to the non-tumor tissue (Figure 1B, Supplementary Figure 1). Furthermore, qRT-PCR analysis was performed by using 3 primary cultured GSCs cell lines (1210, 105 and 823) compared with differentiated GBM cell line U87 and normal human astrocyte (NHA) as a control. The results demonstrated a significant enriched expression of TTK protein in GBM especially in GSCs (Figure 1C). Similarly, western blot results demonstrated the same trends of TTK expression in GSCs (Figure 1D). As CD133 was the most common stemness marker for GSCs, we used MACS CD133 sorting for the enrichment of CD133^High^ GSCs from glioma spheres. As expected, TTK expression was enriched in GSCs which exhibited a high expression of CD133 surface marker when compared with matched differentiated cells from 1210 glioma sphere lines (Figure 1E). Taken together, TTK was highly expressed in GBM, especially in GSCs, indicating that TTK function was essential for GSCs to maintaining stemness.

### TTK was functionally required for *in vitro* proliferation, self-renewal and *in vivo* tumorigenesis of GSCs

**Figure 2:**
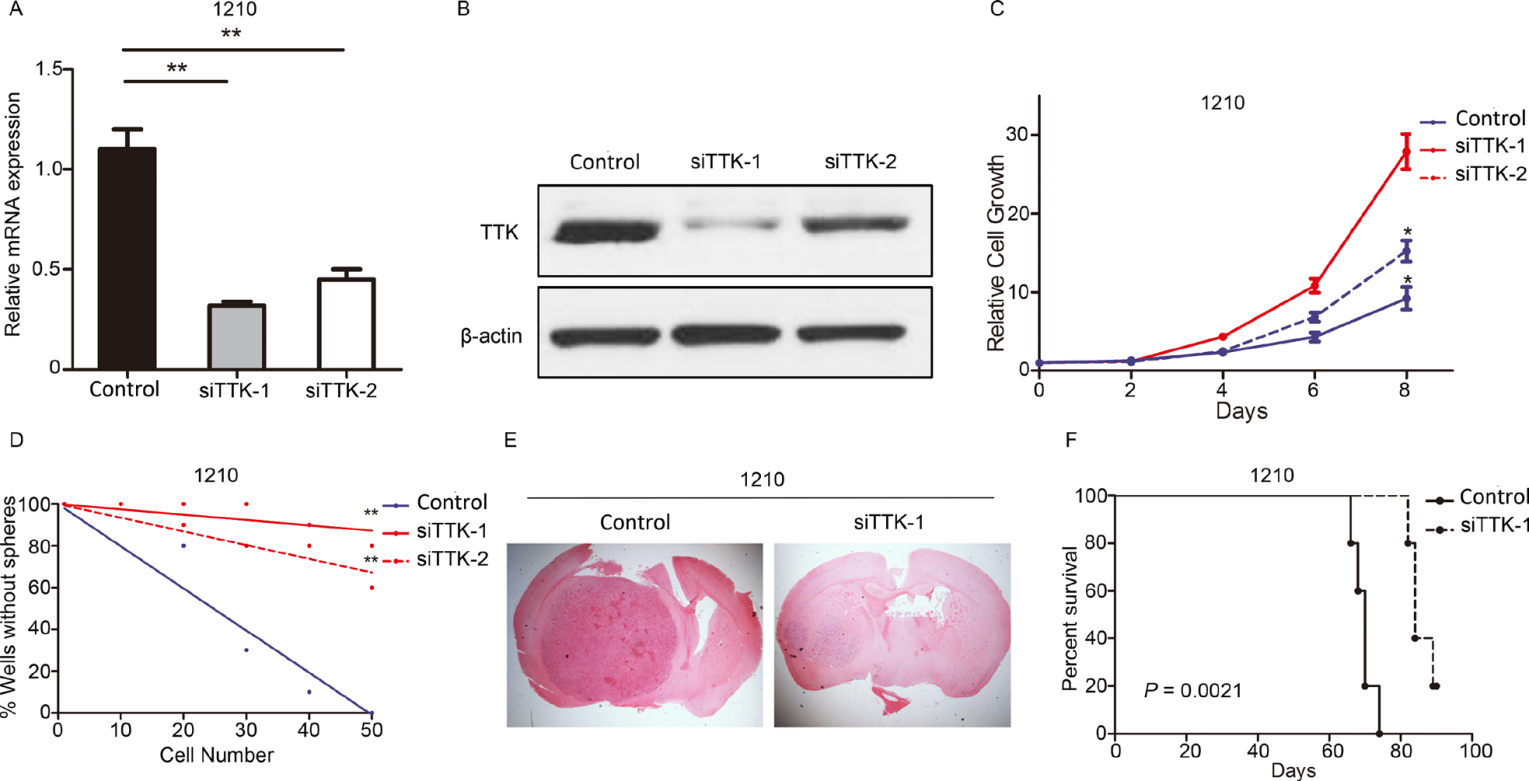
TTK was functionally required for proliferation, self-renewal and *in vivo* tumorigenesis of GSCs (**A**) qRT-PCR analysis of 1210 GSCs transduced with 2 siRNAs against TTK (siTTK-1 and siTTK-2) or control lentivirus (Control). (*n* = 3, ^**^P < 0.01, with one-way ANOVA followed by Dunnett’s post-test). (**B**) Western blot analysis of 1210 GSCs transduced with 2 siRNAs against TTK (siTTK-1 and siTTK-2) or control lentivirus (Control). β-actin served as a control. (**C**) *In vitro* cell growth assay showed siRNAs against TTK (siTTK-1 and siTTK-2) inhibited cell proliferation of 1210 GSCs (*n* = 6, ^*^P < 0.05, with one-way ANOVA). (**D**) An *in vitro* clonogenicity assay by limiting dilution neuro sphere formation indicated that TTK silencing decreased the clonogenicity of 1210 GSCs (^**^P < 0.01, *n* = 10, with ELDA analysis). (**E**) Representative images of H&E stained mouse brain section after the intracranial transplantation of 1210 GSC transduced with 2 siRNAs against TTK (siTTK-1 and siTTK-2) or control lentivirus (Control). (**F**) Kaplan-Meier analysis of nude mice harboring intracranial tumor derived from 1210 GSC transduced with 2 siRNAs against TTK (siTTK-1 and siTTK-2) or control lentivirus (Control) (*n* = 5, *P* = 0.0021, with log-rank test).

To examine the biological role of TTK in GSCs, we picked 2 GSCs (1210 and 823) and transduced with either one of the two lentiviral siRNA clone for TTK (siTTK-1 and siTTK-2) or a negative control lentivirus (Control). qRT- PCR analysis indicated TTK expression was dramatically decreased in siTTK GSCs (Figure 2A, Supplementary Figure 2A). Furthermore, western blot results indicated that TTK protein was markedly decreased by siTTK transfection (Figure 2B, Supplementary Figure 2B). *In vitro* cell growth kinetics of GSCs transfected with siTTK or control lentivirus was inhibited proportionally to the reduction levels of TTK (Figure 2C, Supplementary Figure 2C). To investigate the stemness properties of siTTK-infected GSCs, a limiting-dilution assay was performed with 1210 or 823 glioma spheres infected with siTTK lentivirus (siTTK-1, siTTK-2) or non-targeting lentivirus (Control). The clonal-formation capability of GSCs could be significantly decreased by TTK knock-down (Figure 2D, Supplementary Figure 2D).

Next, to study the effects of artificial TTK knock-down on *in vivo* tumor formation, we picked 2 GSCs (1210 and 823) infected with or without siTTK transfection to establish *in vivo* xenograft mouse intracranial tumor models. The data indicated a more rapid formation of GBM-like tumors in control lentivirus transfected GSCs xenografted mice, consistently accompanied with a shorter survival (Figure 2E, Figure 2F, Supplementary Figure 2E, 2F). Altogether, these results indicated that TTK was functionally required for proliferation and tumorigenesis in GSCs.

### TTK is enriched in GBM and is correlated with poor prognosis in GBM patients

**Figure 3:**
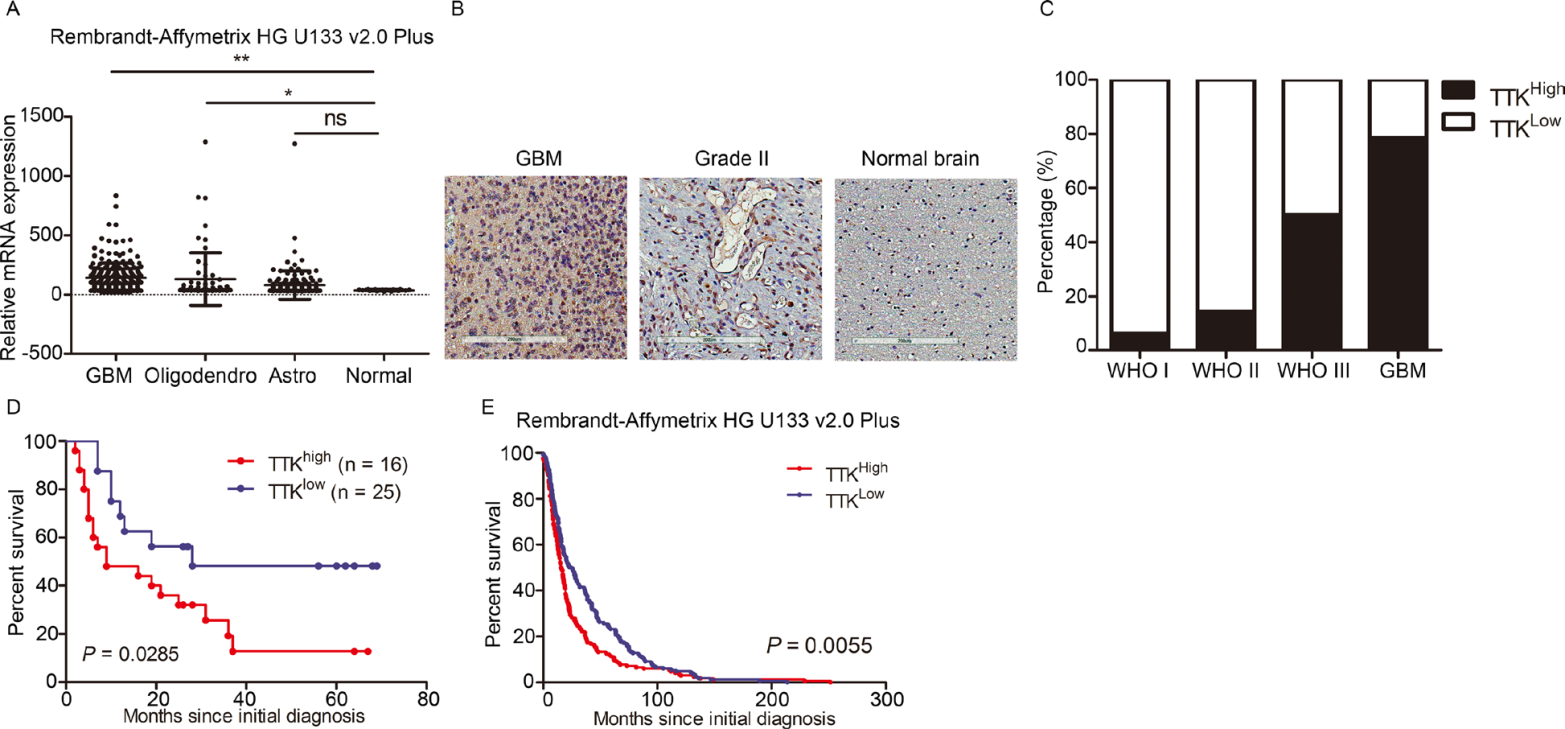
TTK is a clinically relevant molecular target in glioblastoma (**A**) Analysis of Rembrandt database demonstrated that TTK expression in GBM is significantly higher than non-tumor and other types of glioma groups (^*^P < 0.05, ^**^P < 0.01, ns *P* > 0.05, with one-way ANOVA followed by Dunnett’s post-test). (**B**) Representative immunohistochemical images of TTK in glioma samples and non-tumor brain samples. (**C**) TTK expression was elevated in GBM and WHO III glioma samples. (**D**) Kaplan-Meier analysis evaluated the inverted correlation between TTK expression and post-surgical survival of 56 glioma patients (*P* = 0.0285, with log-rank test). (**E**) Kaplan-Meier analysis of the Rembrandt data indicated the inverted correlation between TTK expression and post-surgical survival of GBM patients (*P* = 0.0055, with log-rank test).

Given the results from the previous data, we raised up the question whether TTK was a clinically relevant molecular signature for GBM. An analysis based on Rembrandt database was performed and the results demonstrated that TTK was significantly highly expressed in GBM than non-tumor or other glioma subtypes (e.g. oligodendrocytes and astrocytoma) (Figure 3A). Additionally, we examined TTK expression in 56 glioma tumor tissues and 3 normal brain tissues as negative control by using IHC staining. Higher TTK expression levels were observed in the cytoplasm of glioma cells (Figure 3B). Additionally, German immunohistochemical score (GIS) was used to quantify the expression levels of TTK in each sample. The results found that TTK was highly enriched in GBM samples, however, was much lower in the low grade glioma samples or normal brain tissue (Figure 3C). Furthermore, the overall survival for the TTK^low^ group patients was significantly longer than that in the TTK^high^ group (Figure 3D). Similar results could be achieved when using the Rembrandt database (Figure 3E). Taken together, TTK is likely a specific relevant molecular for GBM, clinically.

### TTK is transcriptionally regulated by MTFR2

**Figure 4:**
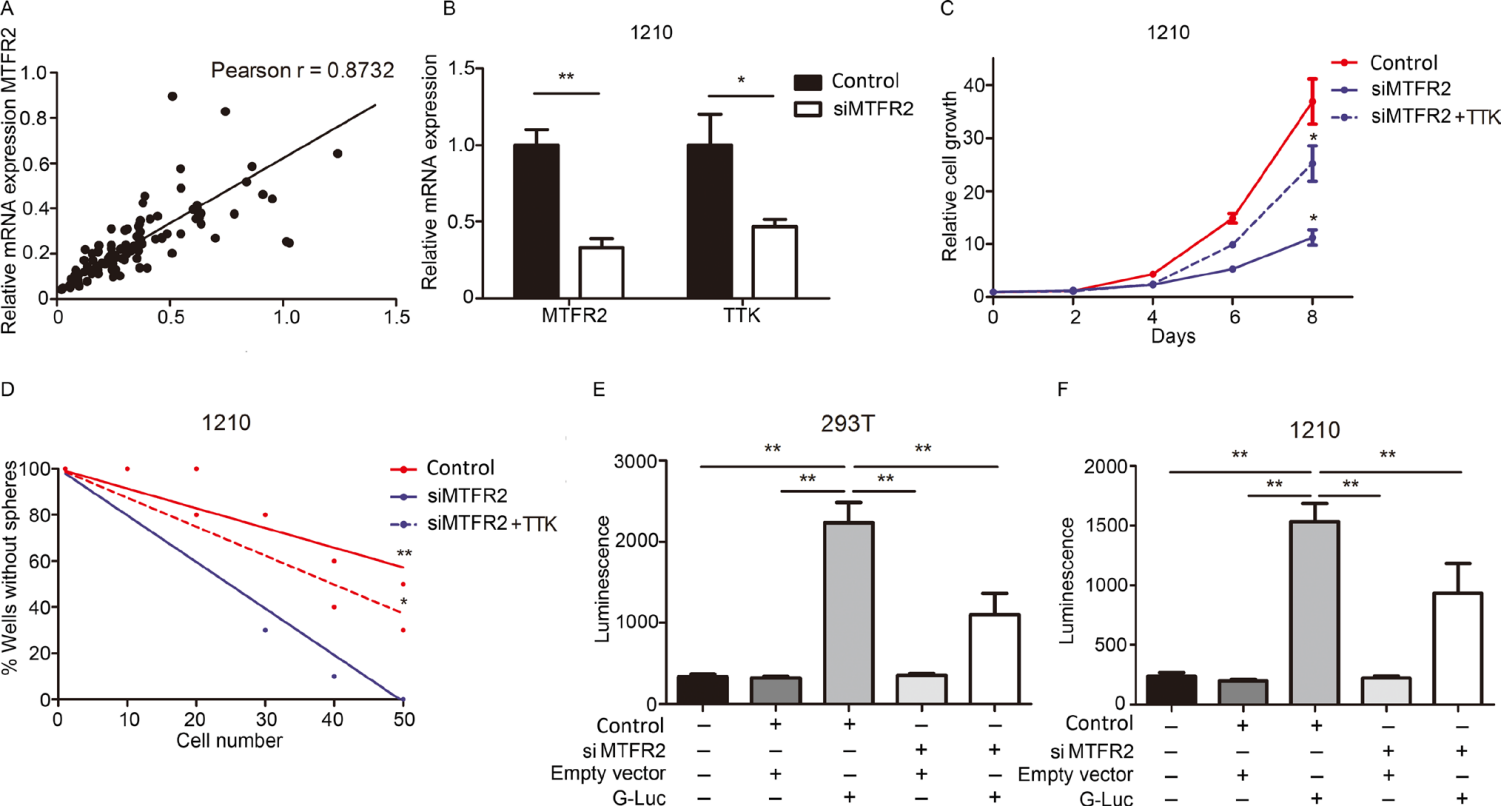
TTK is transcriptionally regulated by MTFR2 (**A**) Person r correlation analysis for TTK and MTFR2 in TCGA database (Person *r* = 0.8732). (**B**) qRT- PCR analysis for MTFR2 and TTK mRNA expression in control lentivirus (Control) or siMTFR2 transfected 1210 GSCs (^*^P < 0.05, ^**^P < 0.01,with *t*-test). (**C**) *In vitro* cell growth assay showed siRNAs against MTFR2 (siMTFR2) inhibited cell proliferation of 1210 GSCs (*n* = 6, ^*^P < 0.05, with one-way ANOVA). (**D**) An *in vitro* clonogenicity assay by limiting dilution neuro sphere formation indicated that MTFR2 silencing decreased the clonogenicity of 1210 GSCs (^*^P < 0.05, ^**^P < 0.01, *n* = 10, with ELDA analysis). (**E**, **F**) Luciferase activity assay showed siRNA-mediated-knockdown of MTFR2 resulted in a marked decrease in transcription activity of MTFR2-binding region on TTK promoter in both 293T cells and 1210 GSCs.

To further assess the regulation mechanism of TTK in GBM, a correlation analysis was performed by using TCGA database. Pearson correlation analysis indicated that MTFR2 was one of the most closely relevant genes for TTK in GBM (Figure 4A, complete results could be found in Supplementary Table 1). Expression data from Rembrandt database indicated that MTFR2 was significantly elevated in GBM samples when compared to other subtypes of glioma or normal brain tissue (Supplementary Figure 3A) and higher MTFR2 expression could be correlated with poor prognosis (Supplementary Figure 3B). Moreover, we treated 1210 GSCs with either sphere culture medium or 10% FBS containing medium for 4 weeks then qRT-PCR was performed. The results indicated that both TTK and MTFR2 were significantly decreased in differentiated cells compared to the naive populations (Supplementary Figure 3C).

To clarify the molecular mechanism between MTFR2 and TTK, an artificial suppression of MTFR2 through lentivirus transfection was used. qRT-PCR analysis indicated that TTK mRNA expression was dramatically reduced in siMTFR2 GSCs (Figure 4B). *In vitro* cell growth kinetics of GSCs was diminished by siMTFR2 transfection (Figure 4C). Furthermore, a limiting-dilution assay indicated that clonal-formation capability was substantially attenuated by MTFR2 knock-down (Figure 4D).

To deeply investigate the MTFR2-dependent regulation mechanism of TTK in GSCs, we performed luciferase assay to measure the TTK promoter activity with reduced expression of MTFR2 in HEK293T cells or 1210 GSCs (Supplementary Figure 4). As expected, siRNA-mediated-knockdown of MTFR2 caused a marked reduction in transcription activity of TTK promoter in both HEK293T cells or 1210 GSCs (Figure 4E, 4F). These data suggest that the activity of TTK promoter is, at least partly, regulated by MTFR2 and this mechanism is critical for TTK expression in GBM and GSCs.

## DISCUSSION

Accumulating data has indicated that cancer stem cells are critical for tumor growth, therapy resistance and recurrence in multiple types of cancer [17, 18]. In GBM patients, the existence of GSCs promotes tumor growth depending on the self-renewal capability and multiple differentiation potential [6]. Recent studies have proved that GSCs gained resistance after radiotherapy and chemotherapy and a wide range of pathways contributed to this phenotype changing [19, 20]. Accumulating evidence suggests that kinase activity are strongly associated to malignancy of GBM and inhibition of kinase-dependent pathways by either inhibitor or targeted immunotherapy exhibits efficient reduction of tumor proliferation and prolonged survival periods in GBM patients [21–23]. Regarding these findings, we identified TTK as one of the most up-regulated and differentially expressed kinase encoding genes in GSCs and was functionally required for *in vitro* clonogenicity and *in vivo* tumor propagation of GSCs. TTK (or MPS1) is a highly conserved dual specificity kinase first identified in yeast and specifically phosphorylate tyrosine and serine/threonine residues [24]. TTK participates cell mitosis mainly through the regulation of kinetochore localization and the spindle assembly checkpoint (SAC), thus, promotes cell growth and migration in multiple types of malignant cancer [11, 24]. Herein, we found that silencing of TTK by siRNAs in GSCs markedly decreased cell growth and tumorigenesis of GSCs, therefore, indicating that TTK might be a new therapeutic target for GBM treatment. Stratford, *et al.* [25] proved that selective inhibition of TTK by small molecules resulted in override of the SAC-induced cell cycle arrest, then reduced the proliferation of pancreatic cancer cells by inducing lethal chromosomal instability. Moreover, 2 novel TTK inhibitors BAY 1161909 and BAY 1217389, which are currently under phase I clinical trials, showed impressive antitumor activity [26]. These TTK specific small molecule inhibitors exhibited fantastic effects to enhance the efficacy of antimitotic cancer drugs while reduced the appearance of drug resistance [26]. Nonetheless, TTK promotes GSCs proliferation whether depending on its functions on mitosis regulation or other unknown mechanism (e.g. stemness maintaining) participates in this process still remains unclear. Further studies are needed to clarify these concerns.

Since TTK is essential for GSCs maintaining in GBM, leading us to look into a more in-depth mechanism study of TTK regulation and activation in GSCs. Recent study indicates that HLF-mediated miR-132 targets on and suppresses TTK expression, thus inhibited cell proliferation, migration and therapy-resistance of glioma cells [27]. Another study indicates that a formation of VDAC3-TTK complex could be observed at the centrosome and induces disassembly of ciliary then suppresses cilia assembly in cells mitotic cycles [28]. However, the transcriptional regulation of TTK is still ambiguous. A genome-wide Pearson correlation analysis by using TCGA database was performed to identify the potential up-stream regulator of TTK. MTFR2 was identified as one of the most correlated genes to TTK and regulated the transcriptional regulation of TTK via activation of TTK promoter. This novel finding implied the direct transcriptional regulating of TTK by MTFR2 through activate TTK promoter. Nonetheless, the specific binding region between MTFR2 and TTK promoter still needs to be further tested. Moreover, MTFR2 is poorly studied in GBM and whether its regulation mechanism of TTK is directly binding or some unknown transcription factor is involved still needs to be investigated. A ChIP-PCR or ChIP-sequencing may be useful to address these questions.

In conclusion, TTK is markedly enriched in GSCs and is functionally required for clonogenicity and tumor propagation. Additionally, enriched TTK expression and indicates poor prognosis in GBM patients. Moreover, MTFR2-dependent regulation of TTK is essential for GSCs maintaining in GBM. Further understanding of the mechanism of TTK and MTFR2 in GSCs may provide new prospects for improving GBM treatment.

## MATERIALS AND METHODS

### Ethics

All the usage of experimental animals and patient samples in this study are approved by the Scientific Ethics Committee of The First Affiliated Hospital of Xi’an Jiaotong University, Xi’an, China.

### Reagents and antibodies

Following reagents and primary antibodies are used in this study: DMEM-F12 (Gibco, 10565-018), Fetal bovine serum (Gibco, 10082-147), Accutase solution (Sigma, A6964-100), Alamar Blue (Invitrogen, DAL1100), RIPA buffer (Sigma, R0278), Phosphatase inhibitor cocktail (Sigma, P0044), Protease inhibitor cocktail (P8340), Bradford (BIORAD, 500-0006), BSA used in Bradford assay (BioLabs, B9001S), PageRuler plus prestained protein (Thermo scientific, 26619), iScript Reverse Transcription supermix for qRT-PCR (Bio-rad, 170-8841), siTTK lentivirus particles (piLenti-siRNA-GFP, abm INC, iV026337), siMTFR2 lentivirus particles (piLenti-siRNA-GFP, abm INC, iV007362), TTK promoter reporter clones (GeneCopoeia, HPRM37034), TTK overexpression lentivirus particles (pLenti-GIII-CMV, abm INC, BC000633), Secrete-Pair Gaussia Luciferase Assay Kit (GeneCopoeia, LF061), anti-TTK andibody (Aviva Systems Biology, OAAF01564, Rabbit), anti-MTFR2 andibody (Novus Biologicals, NBP1-84967, Rabbit), anti-β-actin andibody (Sigma, A5316, Mouse).

### Cell cultures

GSCs 1210, 823 and 105 were established and cultured in Xi’an Jiaotong University following the previous protocols [16]. Experiments with neuro spheres were performed with lines that were cultured for less than 40 passages since their initial establishment. Glioblastoma cells U87 and HEK-293T cells are provided by Xi’an Jiaotong University. Differentiated cell lines are cultured in DMEM/F12 medium containing 10% FBS supplement (vol%). The culture medium is replaced every 5–10 days. Normal Human Astrocytes (NHA, Lonza) are used as a control sample in this study. *In vitro* cell growth assay and sphere formation assay were performed following the previous protocols [3, 29].

### RNA isolation and quantitative Real-Time PCR

RNA is isolated by using RNeasy mini kit (QIAGEN) according to the manufacturer’s instructions. RNA concentration is determined using a Nanodrop 2000 (Thermo scientific). cDNA is synthesized by using iScript reverse transcription supermix for qRT-PCR (Bio-rad) according to the manufacturer’s protocol. The reverse-transcribed cDNA is analyzed by quantitative RT-PCR (qRT-PCR), and 18S is used as an internal control. Each qRT-PCR includes a 10 μL reaction mixture per well that includes 2.5 μL cDNA, 0.5 μL forward primer (0.5 μM), 0.5 μL reverse primer (0.5 μM), 1.5 μL of DNase/RNase-free distilled water, and 5 μL SYBR green reagent (QIAGEN). The following cycles are performed during DNA amplification: 94°C for 2 min, 40 cycles of 94°C (30 s), 60°C (30 s), and 72°C (40 s). 18S is used as an internal control. The primer sequences are showed below: TTK-Forward: GATTGCCACTGTTTCTGGTT, TTK-Reverse: AACCCTGAAGAATAAAACGGA, MTFR2-Forward: GAAACTGGATCCCAATGTGAA, MTFR2-Reverse: GAATAAGGTTAAGCTTCGTGCAA, CD133-forward: ACTCCCATAAAGCTGGACCCC, CD133-reverse: TCAATTTTGGATTCATATGCCTT, 18S-Forward: GGCCCTGTAATTGGAATGAGTC and 18S-Reverse: CCAAGATCCAACTACGAGCTT.

### Western blot

The cell lysates are prepared in RIPA buffer containing 1% protease and 1% phosphatase inhibitor cocktail (Sigma Aldrich) on ice. The sample protein concentrations are determined by the Bradford method. Equal amounts of protein lysates (10 µg/lane) are fractionated on NuPAGE Novex 4–12% Bis-Tris Protein gel (Invitrogen) and transferred to a PVDF membrane (Invitrogen). Subsequently, the membranes are blocked with 5% skimmed milk for 1 h and then treated with the relevant antibody at 4°C overnight. Protein expression is visualized with Amersiam ECL Western Blot System (GE Healthcare Life Sciences). β-actin serves as a loading control.

### MACS CD133 sorting

Cells in 1210 glioma spheres showing high and low expression level of CD133 were separated by MACS method according to manufacturer’s instruction. In brief, single cell suspensions were prepared with Accutase. After 30min incubation with CD133 MicroBeads (Miltenyi Biotec) and FcR blocking reagent at 4°C, the cells were adding into LS columns (Miltenyi Biotec) to get CD133 high and CD133 low cell population. After sorting, the expression level of CD133 in two cell population was confirmed immediately by qRT-PCR.

### Pearson r correlation analysis

Expression data of related genes was extracted from TCGA database. All the data was converted into Log_2_ form and then Pearson r coefficient was calculated by using the following formula and the statistical significance was calculated by using *F*-test.

$$r_{x,y}=\frac{\sum \left(x-\bar{x}\right)\left(y-\bar{y}\right)}{\sqrt{\sum_{i=1}^{n}{\left(x_{i}-\bar{x}\right)}^{2}}\sqrt{\sum_{i=1}^{n}{\left(y_{i}-\bar{y}\right)}^{2}}}$$

### Lentivirus infection

Lentivirus infection was performed following the previous protocols [3, 29]. GFP was used to evaluate the efficacy of lentivirus infection.

### Promoter activity assay

TTK promoter luciferase reporter was transfected into HEK293T or 1210 cells according to the manufacturer’s instructions. Promoter activity assay was evaluated by luciferase activity by using Secrete-Pair Gaussia Luciferase Assay Kit (GeneCopoeia, LF061) according to the manufacturer’s instructions.

### Immunohistochemistry

Immunohistochemistry was performed following the previous protocol [3]. GIS was used to evaluate the expression level of TTK. Percentage of positive cells is classified as 0 (negative), 1 (up to 10%), 2 (11% to 50%), 3 (51% to 80%), or 4 (>80% positive cells), staining intensity is classified as 0 (no staining), 1 (weak), 2 (moderate), or 3 (strong). The final immunoreactivity score is defined as the multiplication of both grading results (percentage of positive cells × staining intensity).

### *In vivo* intracranial xenograft tumor models

6-week-old female nude mice are used for GBM intracranial implantation. All animal experiments are carried out in Xi’an Jiaotong University. The GSCs suspension (1 × 10^5^ cells in 5 μl of PBS) transduced with non-target or siTTK lentivirus is injected into the brains of nude mice after anesthesia. At least six mice are used for each group. Mice were monitored once a day for symptoms related to tumor growth including an arched back, unsteady gait, paralysis of legs and body weight loss. Mice were euthanized by over-does anesthesia of ketamine and xylazine after a total body weight loss of 40% or severe symptoms were observed.

### Statistical analysis

All the data are presented as Mean ± SD. The number of replicates for each experiment is stated in the figure legend. Statistical differences between two groups are evaluated by two tailed *t*-test. The comparison among multiple groups are performed by one-way analysis of variance (one-way ANOVA) followed by Dunnett’s post-test. The statistical significance of Kaplan-Meier survival plot is determined by log-rank analysis. Statistical analysis is performed by Graphpad Prism 6.0, unless mentioned otherwise in figure legend. *P* < 0.05 is considered as statistically significant.

## SUPPLEMENTARY MATERIALS FIGURES AND TABLE




