# Supplementary material for: Targeting dual specificity protein kinase TTK attenuates tumorigenesis of glioblastoma
Source: Oncotarget. 2017 Dec 11;9(3):3081–8. doi: 10.18632/oncotarget.23152 (PMC5790447; doi:10.18632/oncotarget.23152)
Supplement: Supplementary file 1 [file oncotarget-09-3081-s001.pdf]

Targeting dual specificity protein kinase TTK attenuates tumorigenesis of glioblastoma

SUPPLEMENTARY MATERIALS

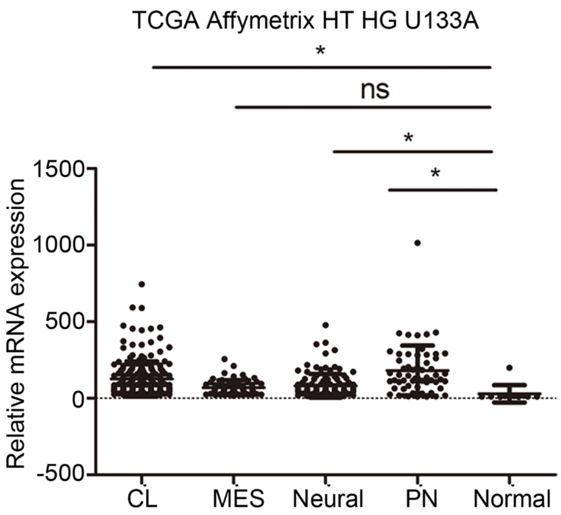

Supplementary Figure 1: Analysis of TCGA database indicated that TTK was highly expressed in all the 4 subgroups of GBM. Classical (CL); Mesenchymal (MES); Neural and Proneural (PN), $^{*}P < 0.05$, $^{**}P < 0.01$, ns $P > 0.05$, with one-way ANOVA followed by Dunnett's posttest, platform Affymetrix HT HG U133A.

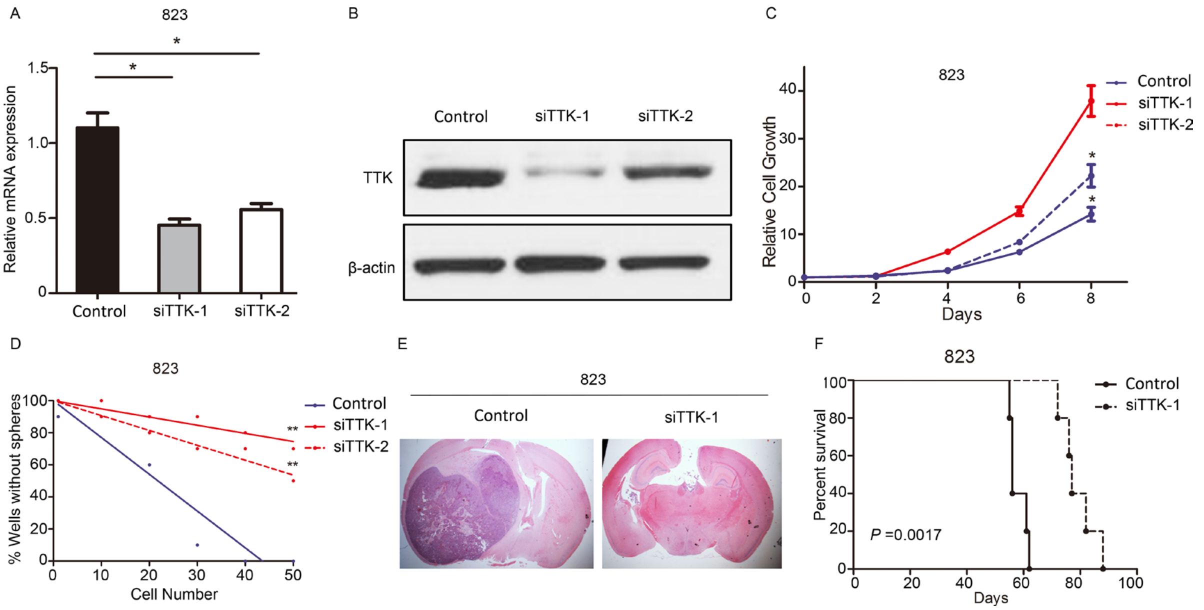

Supplementary Figure 2: TTK was functionally required for proliferation, self-renewal and *in vivo* tumorigenesis of 823 GSCs. (**A**) qRT-PCR analysis of 823 GSCs transduced with 2 siRNAs against TTK (siTTK-1 and siTTK-2) or control lentivirus (Control). ($n = 3$, $^{*}P < 0.05$, with one-way ANOVA followed by Dunnett's posttest). (**B**) Western blot analysis of 823 GSCs transduced with 2 siRNAs against TTK (siTTK-1 and siTTK-2) or control lentivirus (Control). β-actin served as a control. (**C**) *In vitro* cell growth assay siowed siRNAs against TTK (siTTK-1 and siTTK-2) inhibited cell proliferation of 823 GSCs ($n = 6$, $^{*}P < 0.05$, with one-way ANOVA). (**D**) An *in vitro* clonogenicity assay by limiting dilution neuro sphere formation indicated that TTK silencing decreased the clonogenicity of 823 GSCs ($^{**}P < 0.01$, $n = 10$, with ELDA analysis). (**E**) Representative images of H&E stained mouse brain section after the intracranial transplantation of 823 GSC transduced with 2 siRNAs against TTK (siTTK-1 and siTTK-2) or control lentivirus (Control). (**F**) Kaplan-Meier analysis of nude mice harboring intracranial tumor derived from 823 GSC transduced with 2 siRNAs against TTK (siTTK-1 and siTTK-2) or control lentivirus (Control) ($n = 5$, $P = 0.0017$, with log-rank test).

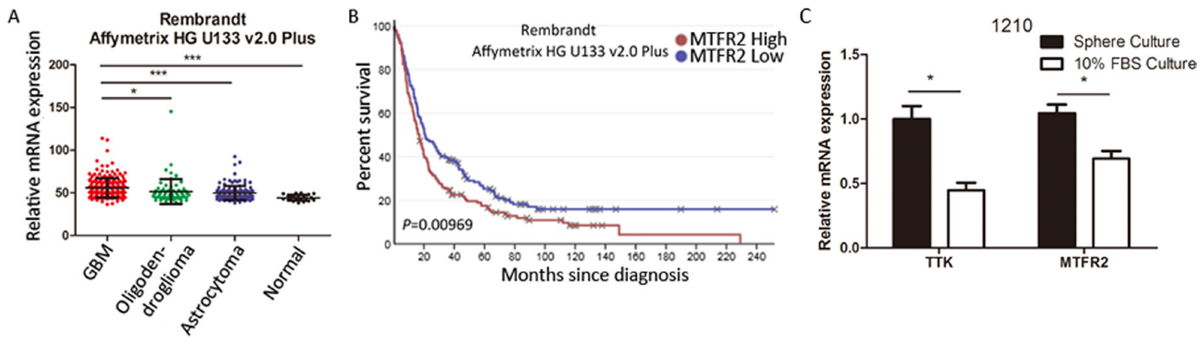

Supplementary Figure 3: MTFR2 was highly expressed in GBM and GSCs. (**A**) Analysis of Rembrandt database demonstrated that MTFR2 expression in GBM is significantly higher than non-tumor and other types of glioma groups ($^{*}P < 0.05$, $^{***}P < 0.001$, with one-way ANOVA followed by Dunnett's post-test). (**B**) Kaplan-Meier analysis of the Rembrandt data indicated the inverted correlation between MTFR2 expression and post-surgical survival of GBM patients ($P = 0.00969$, with log-rank test). **(C)** qRT-PCR analysis showed TTK and MTFR2 expression was decreased in serum treated 1210 GSCs ($n = 3$, $^{*}P < 0.05$, with one-way ANOVA followed by Dunnett's post-test).

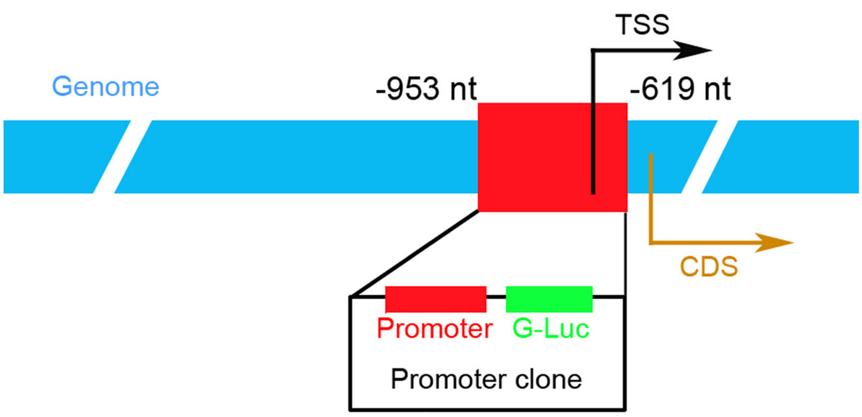

Supplementary Figure 4: Schematic drawing of the promoter region of the human TTK.

Supplementary Table 1: Pearson correlation analysis for TTK in GBM (TCGA). See Supplementary_Table_1
